# Structural magnetic resonance imaging demonstrates volumetric brain abnormalities in down syndrome: Newborns to young adults

**DOI:** 10.1016/j.nicl.2021.102815

**Published:** 2021-09-03

**Authors:** Bernadette McCann, Jacob Levman, Nicole Baumer, Melanie Y. Lam, Tadashi Shiohama, Liam Cogger, Allissa MacDonald, Prahar Ijner, Emi Takahashi

**Affiliations:** a Department of Human Kinetics, St. Francis Xavier University, Antigonish, NS B2G 2W5, Canada; b Department of Computer Science, St. Francis Xavier University, Antigonish, NS B2G 2W5, Canada; c Department of Neurology, Boston Children’s Hospital, 300 Longwood Ave, Boston, MA 02115, USA; d Department of Pediatrics, Graduate School of Medicine, Chiba University, Chiba, Japan; e Department of Education, St. Francis Xavier University, Antigonish, NS B2G 2W5, Canada; f Department of Biology, St. Francis Xavier University, Antigonish, NS B2G 2W5, Canada; g Division of Newborn Medicine, Department of Medicine, Boston Children’s Hospital, 401 Park Dr., Boston, MA 02215, USA; h Department of Pediatrics, Harvard Medical School, Boston, MA, USA

**Keywords:** Magnetic resonance imaging, Down syndrome, Entorhinal, Perirhinal, Volumetrics, Clinical

## Abstract

Down syndrome (DS) is a genetic disorder caused by the presence of an extra full or partial copy of chromosome 21 and characterized by intellectual disability. We hypothesize that performing a retrospective analysis of 73 magnetic resonance imaging (MRI) examinations of participants with DS (aged 0 to 22 years) and comparing them to a large cohort of 993 brain MRI examinations of neurotypical participants (aged 0 to 32 years), will assist in better understanding what brain differences may explain phenotypic developmental features in DS, as well as to provide valuable confirmation of prospective literature findings clinically. Measurements for both absolute volumes and volumes corrected as a percentage of estimated total intracranial volume (%ETIV) were extracted from each examination. Our results presented novel findings such as volume increases (%ETIV) in the perirhinal cortex, entorhinal cortex, choroid plexus, and Brodmann’s areas (BA) 3a, 3b, and 44, as well as volume decreases (%ETIV) in the white matter of the cuneus, the paracentral lobule, the postcentral gyrus, and the supramarginal gyrus. We also confirmed volumetric brain abnormalities previously discussed in the literature. Findings suggest the presence of volumetric brain abnormalities in DS that can be detected clinically with MRI.

## Highlights

• Volumetric abnormalities detected in the perirhinal, entorhinal memory regions.
• Volumetric abnormalities detected in key additional brain regions.
• Extensive clinical validation of previous Down Syndrome findings included.
• Perirhinal and entorhinal involvement in Alzheimer’s etiology hypothesized.

## 1 Introduction

Down syndrome (DS) is the most common chromosomal disorder with a prevalence of 8.27 per 10,000 in the United States (Presson et al., 2013). There are three types of DS: trisomy 21, translocation DS, and mosaic DS (Shin et al., 2010). Trisomy 21 is characterized by each cell in the body having three copies of chromosome 21 (Shin et al., 2010, Martin et al., 2009, Roberts et al., 2007). Translocation DS is characterized by chromosome 21 being attached to another chromosome (Shin et al., 2010). Mosaic DS is characterized by the presence of three copies of chromosome 21 occurring in some, but not all cells (Shin et al., 2010, Lowry et al., 1976, Nielsen and Sillesen, 1975, Sherman et al., 2007).

The overexpression of genes on chromosome 21 produces a well-recognized phenotype with abnormalities in both physical and cognitive development (Silverman, 2007). Cognitive deficits include reduced memory (Carlesimo et al., 1997, Chapman and Hesketh, 2001, Jarrold et al., 2008), learning deficits (Fidler and Nadel, 2007, Wan et al., 2017), and difficulties in speech and language (Abbeduto et al., 2007, Jacola et al., 2014). People with DS are at an increased risk for multiple health problems such as congenital heart disease, hearing loss, ophthalmological problems, psychiatric disorders and Alzheimer’s disease (AD) (Ropper and Bull, 2020, Roizen and Patterson, 2003).

Although people with DS are at increased risk for multiple health conditions, their quality of life and life expectancy has continued to increase (Brown et al., 2001). In 1929, life expectancy was just 9 years, whereas in 2010 life expectancy approached 60 years (Torr et al., 2010). In Australia, life expectancy has increased from 18 years in 1963 to 60 years in 2002 (Bittles and Glasson, 2004), a pattern which is reflected in the United States (Presson et al., 2013). These increases in life expectancy may be due to improvements in general and medical care (Torr et al., 2010), earlier treatments of congenital heart disease and respiratory infections (Coppus, 2013), and increased community living rather than institutional care (Bittles et al., 2007). Additional research on the underlying causes of DS may lead to more effective interventions and treatments, and further improvements in quality of life and longevity.

Researchers have used neuroimaging techniques to investigate abnormalities in brain development to better understand the neurological underpinnings of developmental challenges and progression in DS. Magnetic resonance imaging (MRI) is an imaging technology used to form detailed three-dimensional anatomical and physiological images. Performing MRI in populations with specific physical and cognitive abnormalities can help establish possible associations between the presentation of specific brain structures and potentially associated behavioral symptoms. Automated MRI brain analysis tools (e.g. Fischl, 2012) can assess volumetric brain measurements by differentiating between white matter (WM), gray matter (GM), and cerebrospinal fluid (CSF) while relying on statistical atlases of neuroanatomy.

Multiple MRI studies have been conducted to gain a better understanding of the presentation of the brains of individuals with DS. Several neuroimaging studies have reported total brain volume reductions (Aylward et al., 1999, Aylward et al., 1997, Beacher et al., 2010, Frangou et al., 1997, Kates et al., 2002, Menghini et al., 2011, Pearlson et al., 1998, Pinter et al., 2001a, Pinter et al., 2001b, Śmigielska-Kuzia et al., 2011, Weis et al., 1991). Studies have also reported specific volume reductions in the cerebrum (Jernigan et al., 1993, Jernigan and Bellugi, 1990, Kates et al., 2002, Raz et al., 1995), cerebellum (Beacher et al., 2010, Carducci et al., 2013, Jernigan et al., 1993, Jernigan and Bellugi, 1990, Pinter et al., 2001b, Raz et al., 1995, Wang et al., 1992, Weis et al., 1991, White et al., 2003), hippocampus (Aylward et al., 1999, Carducci et al., 2013, Pearlson et al., 1998, Pinter et al., 2001a, Raz et al., 1995, Śmigielska-Kuzia et al., 2011, Teipel et al., 2003), brainstem (Carducci et al., 2013, Fujii et al., 2017, Jernigan and Bellugi, 1990), and frontal lobes (Beacher et al., 2010, Carducci et al., 2013, Sabbagh et al., 2015, Śmigielska-Kuzia et al., 2011, White et al., 2003). Increases in the volume of the lateral ventricles (Beacher et al., 2010, Frangou et al., 1997, Pearlson et al., 1998, White et al., 2003) and the third ventricle (Raz et al., 1995) have also been reported. More recently, studies have extended these findings to fetal imaging and demonstrated abnormal fetal brain development in the cortex and cerebellum in DS (Tarui et al., 2020, Patkee et al., 2020).

In this study, we hypothesize that comparison of volumetric measurements in various brain regions in individuals with DS to those without DS across age groups up to 20 years may help identify regional abnormalities not detected in previous studies, as well as to clinically validate findings observed in previous research.

## 2 Materials and methods

### 2.1 Participants

This subsection of the methods was previously completed as part of an earlier analysis on Down Syndrome, focused on cortical thicknesses in grey matter (Levman et al., 2019), whereas this manuscript is focused on volumetric regional biomarkers that include the white matter, the ventricles, the choroid plexuses as well as the grey matter. Following approval by Boston Children’s Hospital’s (BCH) Institutional Review Board (who waived the need of informed consent due to lack of risk to participants in this retrospective analysis), the BCH clinical imaging electronic database was reviewed from 01/01/2008 until 02/24/2016, and all brain MRI examinations of participants aged 0 to 32 years were included for further analysis if DS was indicated in the participant’s electronic medical records. Examinations deemed low quality (due to excessive participant motion, large metal artifact from dental hardware, lack of a T1 structural imaging volume providing diagnostically useful axial, sagittal and coronal oriented images, etc.) were excluded from this analysis. Examinations that were inaccessible due to technical reasons were also excluded. This generated a total of 73 examinations from DS participants. 73% of our DS examinations included patients with congenital heart defects, according to their medical records. The neurotypical cohort was assembled retrospectively in a previous analysis (Levman et al., 2017), where participants were selected on the basis of normal MRI examinations, as assessed by a BCH neuroradiologist, and medical records with no indication of any neurological problems (participants with a known disorder such as autism, cerebral palsy, traumatic brain injury, developmental delay, tuberous sclerosis complex, stroke, neurofibromatosis, epilepsy, attention deficit hyperactivity disorder, etc. were excluded). Participants with cancer were also excluded in order to avoid data exhibiting growth trajectories that are affected by treatments such as chemotherapy. The exclusion criteria used for the DS participants was also applied to the neurotypical participants yielding 993 examinations. Demographic information on studied participants is presented in Table 1, the information therein having been previously presented (Levman et al., 2019)**.**

**Table 1:** Demographic information on study participants with hemispheric and whole brain group-wise comparisons.

Demographic measures and comparative statistics	0–5 Years	5–10 Years	10–15 Years	15–20 Years
DS mean age (std dev) in years	2.12 (1.25)	7.33 (1.30)	13.78 (0.69)	16.35 (0.86)
Healthy mean age (std dev) in years	2.59 (1.43)	7.63 (1.41)	12.41 (1.41)	16.70 (1.11)
DS age range in years	0.62–4.71	5.17–9.65	12.15–14.86	15.16–17.45
Healthy age range in years	0.00–4.99	5.02–9.98	10.04–14.99	15.01–19.95
DS male/female count	17 / 9	12 / 10	10 / 5	7 / 2
Healthy male/female count	71 / 68	124 / 137	115 / 177	80 / 194
Comparative total cortical volume, Cohen’s d statistic	−0.62	−0.89	−0.48	−0.17

### 2.2 MRI data acquisition and preprocessing

This subsection of the methods was previously completed as part of an earlier analysis on Down Syndrome, focused on cortical thicknesses in grey matter (Levman et al., 2019). All participants (both DS and neurotypical) were imaged with clinical 3 T MRI scanners (Skyra, Simens Medical Systems, Erlangen, Germany) at BCH yielding T1 structural volumetric imaging examinations which were accessed through the Children’s Research and Integration System (Pienaar et al., 2014). There is variability in the pulse sequences employed to acquire these volumetric T1 examinations due to the clinical and retrospective nature of this study, with spatial resolution in the x and y directions varying from 0.2 to 1.4 mm (0.9 mm on average) and through plane thickness varying from 0.5 to 2 mm (1 mm on average). Strengths and limitations of the large-scale varying MR protocol approach used in this study are addressed in the Discussion. A single volumetric MRI was acquired from each imaging session, with some patients returning for multiple MRI examinations (different imaging sessions) which were used in the analysis. Motion correction was not performed, but examinations were visually assessed and those with substantial motion artifacts were excluded. T1 structural examinations were processed with FreeSurfer (Fischl, 2012), using the recon-all command to align the input examination to all available brain atlases. Those atlases that include volumetric measurements were included for further analysis (atlases: aseg, aparc, aparc.a2009s, aparc.DKTatlas40, BA, BA.thresh, entorhinal_exvivo, wmparc). These combined atlases include definitions of 232 brain regions from which volumetric measurements were extracted. Each FreeSurfer output T1 structural examination was displayed with label map overlays and was visually examined for quality of regional segmentation results. Exams were excluded from this analysis if FreeSurfer results were observed to substantially fail (i.e. FreeSurfer regions-of-interest (ROIs) that did not align to the MRI and examinations where major problems were observed with an ROI such as a cerebellar segmentation extending far beyond the extent of the cerebellum).

In our DS cohort, these criteria resulted in the exclusion of 1 exam due to a segmentation error, 36 due to technical problems accessing examinations, 65 due to lack of available volumetric examination (thus being incompatible with FreeSurfer technology), 1 due to no non-contrast enhanced volumetric exam, 1 due to a motion artifact and 31 due to FreeSurfer’s failure to complete execution on the patient’s exam. Thus, our final inclusion of 73 examinations represents 35% of all DS MRI examinations available. In our healthy cohort, 58 exams were excluded due to FreeSurfer’s failure to complete execution on the patient’s exam, 1 due to major motion artifact, 1 due to an imaging artifact, 231 due to lack of volumetric examination, 7 due to no non-contrast enhanced volumetric exam and 20 due to technical problems accessing the examinations. The DS group had considerably higher rates of exclusions, which is likely related to the additional challenges in successfully imaging this cohort. The overall rates of motion artifacts are low in both groups, because at BCH, the MR technicians repeat an additional structural MRI examination when motion artifacts are observed. Thus, imaging sessions produce 1–3 volumetric examinations per patient, one of which was selected for this study based on imaging quality.

### 2.3 Statistical analysis

This study included the acquisition of 463 regionally distributed volumetric measurements per imaging examination, as extracted by FreeSurfer’s recon-all command which processes the input examination with all available brain atlases (Fischl, 2012). This included all regional volumetric measurements available such as whole brain measurements, whole hemisphere measurements, ventricular measurements as well as regional white and gray matter measurements in order to perform as thorough an assessment as possible. Participants were divided into four different age groups: early childhood (0–<5 years old), late childhood (5–<10 years old), early adolescence (10–<15 years old) and late adolescence (15–20 years old). We had very few participants over the age of 20 years and so we did not include them in a separate group. We were interested in assessing the extent of group-wise differences of these clinically acquired measurements and so we compared each FreeSurfer extracted measurement within each age range in a group-wise manner (DS compared with neurotypical) with Cohen’s d statistic (positive/negative values indicate a higher/lower average value in the DS population relative to the neurotypical population). Cohen’s d statistic was selected because it is the most established method to assess effect sizes. For each comparison, a p-value based on the standard *t*-test (Student, 1908) for two groups of samples was also calculated. This calculation yielded a total of m = 1,852 group-wise comparisons which led to a Bonferroni corrected threshold for achieving statistical significance of p < 0.05/m = 2.70e^−5^.

To confirm that the findings reported are a result of group-wise differences between the DS and typically developing participants, a statistical model based on multivariable regression (MATLAB R2018a, MathWorks Inc., MA, USA) was constructed, adjusting each measurement within each age range to control for group-wise differences in age, gender, and estimated total intracranial volume. Age and estimated total intracranial volume were treated as continuous variables by the multivariable regression model, within each age range considered. This model was used to adjust each volumetric measurement, in order to evaluate whether the group-wise differences observed between our DS and neurotypical populations are the result of age, gender, or intracranial volume effects.

## 3 Results

Many brain regions showed Bonferroni-corrected, statistically significant differences in volumetric measurements between DS participants and neurotypical controls, with leading absolute volume measurements summarized in Table 2 and leading volumetric findings as percentages of estimated total intracranial volume (%ETIV) summarized in Table 3. Of the 1,852 group-wise comparisons performed, 10.2% exceeded the Bonferroni-correction for statistical significance. All age groupings and left and right hemisphere results (when available) are provided for ease of comparison with each measurement in Table 2 and Table 3 exceeding the Bonferroni correction in at least one age group on at least one corresponding atlas measurement as indicated by bolded entries in the table. Our analysis included four age cohorts (0–5, 5–10, 10–15, and 15–20 year olds), and a general trend was observed whereby the youngest age cohort (0–5 year olds) tended to exhibit the smallest effect sizes (d statistics closest to zero). The leading measurements of interest identified in Table 2, Table 3 have potential to be associated with known symptoms of DS, and is addressed in detail in the Discussion.

**Table 2:** Age-dependent analysis – Leading absolute volumetric measurements sorted by effect size (Cohen’s d statistic).

**Region name**	**Ages 0**–**5 years L&R: d**	**Ages 5**–**10 years L&R: d**	**Ages 10**–**15 years L&R: d**	**Ages 15**–**20 years L&R: d**	**MAX ABS(d)**
Inferior lateral ventricle	**L(1.4946) R(0.91509)**	**L(1.5779) R(1.3824)**	**L(3.2728) R(2.2679)**	**L(2.3108) R(1.8515)**	3.272842451
Lateral ventricle	**L(1.4154) R(1.2448)**	**L(1.0531)** R(0.68878)	**L(2.8718) R(1.4104)**	**L(1.5724)** R(0.94024)	2.871804755
Cerebellum WM	**L(−0.94604) R(−1.1902)**	**L(−1.4894) R(−1.8025)**	**L(−1.5258) R(−1.8775)**	**L(−1.8722) R(−2.1152)**	2.115180421
Cerebellar cortex WM	**L(−1.6176) R(−1.6287)**	**L(−2.0299) R(−1.7797)**	**L(−1.841) R(−1.6847)**	**L(−1.9589) R(−1.9042)**	2.029942547
Superior part of the precentral sulcus GM	L(**−**0.31924) R(**−**0.40641)	L(**−**0.22471) R(**−**0.6696)	**L(1.9091)** R(0.29135)	L(0.2486) R(**−**0.56702)	1.909145297
Brainstem	**−1.1564**	**−1.568**	**−1.379**	**−1.741**	1.741029354
Paracentral WM	**L(−0.9054)** R(**−**0.72034)	**L(−1.2141) R(−1.3405)**	**L(−1.4318) R(−1.7084)**	**L(−1.4609) R(−1.5251)**	1.708408681
Choroid plexus	L(0.69619) R(0.68218)	**L(1.2122) R(1.0377)**	**L(1.4943) R(1.6836)**	L(1.0811) **R(1.704)**	1.704033719
Insula WM	L(**−**0.54246) R(**−**0.6554)	**L(−1.4245) R(−1.5638)**	L(**−**1.2209) R(**−**1.0956)	L(**−**1.3208) **R(−1.645)**	1.644979429
Inferior segment of the circular sulcus of the insula GM	L(**−**0.70291) **R(−0.89004)**	**L(−1.1329) R(−1.4049)**	**L(−1.5564)** R(**−**1.2692)	L(**−**1.0502) R(**−**1.2907)	1.556355215
Superior frontal WM	L(**−**0.62528) R(**−**0.77455)	**L(−1.224) R(−1.2099)**	L(**−**0.96694) R(**−**0.94259)	**L(−1.5143)** R(**−**1.2163)	1.514281309
Lateral orbitofrontal WM	L(**−**0.59017) R(**−**0.65456)	**L(−1.5059) R(−1.3181)**	L(**−**0.97118) R(**−**1.0434)	**L(−1.3579)** R(**−**1.1111)	1.505917201
Hippocampus	L(**−**0.54745) R(**−**0.7131)	**L(−1.4825) R(−1.4472)**	**L(−1.3209)** R(**−**0.91546)	L(**−**1.1283) R(**−**1.0191)	1.482503063
Lateral aspect of the superior temporal gyrus GM	L(**−**0.54903) **R(−0.82521)**	**L(−1.3928) R(−1.3383)**	L(**−**1.0053) **R(−1.4716)**	L(**−**0.92286) R(**−**1.0713)	1.471616645
Putamen	L(**−**0.17481) R(**−**0.14853)	L(0.56893) R(0.77608)	L(0.72863) R(0.95416)	L(1.0603) **R(1.4685)**	1.468527288
Rostral anterior cingulate GM	L(**−**0.64489) **R(−0.87917)**	**L(−1.4555) R(−1.2387)**	L(**−**1.0898) R(**−**0.87017)	L(**−**1.1582) R(**−**0.64821)	1.455525439
Parahippocampal gyrus GM	L(**−**0.0034482) R(0.10231)	L(**−**0.11971) R(0.23946)	**L(1.4479)** R(0.49797)	L(1.0169) R(0.59231)	1.447922379
Supramarginal gyrus GM	L(**−**0.50573) R(**−**0.70025)	L(**−**0.45229) R(**−**0.59699)	L(**−**0.87729) **R(−1.4348)**	L(**−**0.30564) R(**−**0.43076)	1.434824279
Superior temporal GM	L(**−**0.59986) **R(−0.90335)**	**L(−1.4336) R(−1.3471)**	L(**−**1.0762) R(**−**1.2548)	L(**−**0.80652) R(**−**0.9651)	1.433586749
Anterior part of the cingulate gyrus and sulcus GM	L(**−**0.77609) R(**−**0.80217)	**L(−1.4288) R(−1.1201)**	L(**−**1.2411) R(**−**0.97951)	L(**−**1.0524) R(**−**0.63078)	1.428842705
Precentral WM	L(**−**0.81477) **R(−0.83052)**	**L(−0.96969) R(−1.0243)**	L(0.076411) R(**−**0.99735)	L(**−**1.0587) **R(−1.4216)**	1.421592865
Superior temporal WM	L(**−**0.6813) **R(−0.82618)**	**L(−1.1381) R(−1.3808)**	L(**−**1.2331) **R(−1.3866)**	L(**−**1.2751) R(**−**1.2908)	1.386649909
Estimated total intracranial volume	−0.63264	**−1.3595**	−0.74553	−1.0516	1.359525481
Transverse temporal GM	L(**−**0.54361) **R(−0.82729)**	L(**−**0.87162) **R(−1.2091)**	**L(−1.3576) R(−1.2763)**	L(**−**0.85351) R(**−**1.1677)	1.357581815
Amygdala	L(**−**0.22923) R(**−**0.31682)	L(0.48276) R(0.60247)	L(1.105) **R(1.3492)**	L(0.46611) R(0.78873)	1.34915885
Postcentral WM	L(**−**0.80764) **R(−0.94511)**	**L(−1.097) R(−1.1509)**	L(**−**1.0759) **R(−1.3114)**	L(**−**1.0716) R(**−**1.2166)	1.311356434
Thalamus	L(**−**0.72224) R(**−**0.74049)	**L(−1.2082)** R(**−**0.75951)	**L(−1.2982)** R(**−**0.76484)	L(**−**1.2453) R(**−**0.98245)	1.298174753
Planum temporale GM	L(**−**0.67024) R(**−**0.57544)	**L(−1.2552)** R(**−**0.87553)	L(**−**0.9296) R(**−**0.39078)	L(**−**0.86722) R(**−**0.36615)	1.255177672
Whole brain without ventricles	−0.71092	**−1.2543**	−1.0173	−0.94989	1.254290835
Cuneus WM	L(**−**0.60031) R(**−**0.53023)	L(**−**0.8888) R(**−**0.92643)	L(**−**1.2266) R(**−**0.36222)	L(**−**1.057) R(**−**1.1287)	1.226570793
Cortical WM	L(**−**0.57015) R(**−**0.65699)	**L(−1.2085) R(−1.1712)**	L(**−**1.084) R(**−**1.0327)	L(**−**1.2116) R(**−**1.1613)	1.208545094
Whole brain	−0.67566	**−1.2079**	−0.89488	−0.86489	1.207881323
Total cortical WM	−0.61818	**−1.1944**	−1.0613	−1.1886	1.194429529
Caudate	L(0.1911) R(0.25015)	L(**−**0.00063788) R(0.084725)	L(1.0128) R(1.1801)	L(0.18445) R(0.64524)	1.18010787
Lateral orbitofrontal GM	L(**−**0.56728) R(**−**0.45552)	**L(−1.1514)** R(**−**0.77386)	L(**−**0.59393) R(**−**0.4269)	L(**−**0.66505) R(**−**0.2446)	1.15137559
Superior parietal WM	L(**−**0.5974) R(**−**0.70984)	**L(−1.0977) R(−1.1513)**	L(**−**0.86698) R(**−**0.54841)	L(**−**0.86573) R(**−**1.0992)	1.151278084
Central corpus callosum	−0.43232	**−1.1512**	−0.99217	−0.99977	1.151230544
Brodmann’s area 6 GM	L(**−**0.62047) R(**−**0.79394)	L(**−**0.87774) **R(−1.1361)**	L(0.29914) R(**−**0.4708)	L(**−**0.50574) R(**−**0.71708)	1.13608718
Fusiform GM	L(**−**0.42756) R(**−**0.49504)	**L(−1.1343) R(−1.0125)**	L(**−**0.61961) R(**−**0.98312)	L(**−**0.45556) R(**−**0.57385)	1.134253903
Anterior transverse temporal gyrus (of Heschl) GM	L(**−**0.42636) R(**−**0.75052)	L(**−**0.78765) R(**−**1.1314)	L(**−**1.0277) R(**−**0.78224)	L(**−**0.53841) R(**−**1.0356)	1.131395233
Total GM	−0.70184	**−1.1076**	−0.79214	−0.54629	1.107586546
Lateral occipito**−**temporal sulcus GM	L(**−**0.36848) R(**−**0.57081)	L(**−**0.88792) **R(−1.0961)**	L(**−**0.6234) R(**−**0.94113)	L(**−**0.63684) R(**−**0.86074)	1.096074018
Banks of the superior temporal sulcus GM	L(**−**0.56911) R(**−**0.73142)	**L(−1.0759)** R(**−**0.86864)	L(**−**0.93724) R(**−**0.93752)	L(**−**0.70887) R(**−**0.47153)	1.075855164
Insula GM	L(**−**0.38226) R(**−**0.60433)	L(**−**0.81122) **R(−1.0526)**	L(**−**0.84888) R(**−**1.128)	L(**−**0.65589) R(**−**1.0412)	1.052584227
Cerebrum	−0.57925	−1.0315	−0.80516	−0.69666	1.031544379
Cerebrum without ventricles	−0.57704	−1.0293	−0.80625	−0.69567	1.02932493
Precuneus WM	L(**−**0.56534) R(**−**0.5922)	L(**−**0.74629) **R(−1.0237)**	L(**−**0.63747) R(**−**0.8257)	L(**−**0.78872) R(**−**0.9299)	1.023719086
Superior temporal sulcus (parallel sulcus) GM	L(**−**0.43361) R(**−**0.70324)	**L(−1.0114) R(−1.0022)**	L(**−**0.46643) R(**−**1.061)	L(**−**0.2233) R(**−**0.55707)	1.011433063
Medial orbitofrontal WM	L(**−**0.45367) R(**−**0.48486)	**L(−0.96892)** R(**−**0.88666)	L(**−**1.0978) R(**−**0.83128)	L(**−**1.1112) R(**−**0.99583)	0.968916045
Pars triangularis WM	L(**−**0.64139) R(**−**0.50908)	L(**−**0.80471) R(**−**0.9547)	L(**−**1.0272) R(**−**0.16099)	L(**−**0.99509) R(**−**0.63724)	0.954699679
Anterior transverse collateral sulcus GM	L(**−**0.51085) R(**−**0.75841)	L(**−**0.94293) R(**−**0.7235)	L(**−**1.0823) R(**−**0.65351)	L(**−**0.87673) R(**−**0.45766)	0.942929892
Superior segment of the circular sulcus of the insula GM	L(**−**0.58043) R(**−**0.73673)	L(**−**0.78131) R(**−**0.91379)	L(**−**1.0597) R(**−**0.83424)	L(**−**0.63671) R(**−**0.49956)	0.913794739
Supramarginal WM	L(**−**0.44668) R(**−**0.68379)	L(**−**0.90674) R(**−**0.76209)	L(**−**1.0523) R(**−**0.73248)	L(**−**1.0335) R(**−**0.79555)	0.906740767
Transverse temporal sulcus GM	L(**−**0.6475) **R(−0.85302)**	L(**−**0.58411) R(**−**0.90334)	L(**−**0.88971) R(**−**0.65104)	L(**−**0.36918) R(**−**0.64198)	0.903339677
Caudal anterior cingulate WM	L(**−**0.59894) R(**−**0.64574)	L(**−**0.63222) R(**−**0.90179)	L(**−**0.91116) R(**−**0.81692)	L(**−**0.79927) R(**−**0.81706)	0.901792812
Orbital gyri GM	L(**−**0.52465) R(**−**0.28956)	L(**−**0.89137) R(**−**0.66851)	L(**−**0.23353) R(**−**0.24742)	L(**−**0.41421) R(**−**0.018396)	0.891370891
Lateral occipital WM	L(**−**0.48812) R(**−**0.46746)	L(**−**0.89077) R(**−**0.83387)	L(**−**0.5842) R(**−**0.52019)	L(**−**0.59719) R(**−**0.46873)	0.890769705
Precentral gyrus GM	L(**−**0.60367) R(**−**0.81263)	L(**−**0.66809) R(**−**0.812)	L(0.020972) R(**−**0.7973)	L(**−**0.31704) R(**−**0.82807)	0.812629499
Middle-anterior part of the cingulate gyrus and sulcus (aMCC)	L(**−**0.80905) R(**−**0.77131)	L(**−**0.80854) R(**−**0.85577)	L(**−**0.96773) R(**−**0.96501)	L(**−**0.77688) R(**−**0.92103)	0.80905138
Frontal pole WM	L(**−**0.61799) R(**−**0.75825)	L(**−**0.60333) R(**−**0.27884)	L(**−**0.73725) R(**−**0.89518)	L(**−**0.71345) R(**−**0.46786)	0.758254228

Abbreviations/Symbols: GM = gray matter; WM = white matter; R = right; L = Left; d = Cohen’s d statistic. Bold entries indicate a statistically significant finding after multiple comparisons correction on at least one available FreeSurfer atlas.

**Table 3:** Age-dependent analysis – Leading volumetric measurements as percentages of estimated total intracranial volume (%ETIV) sorted by effect size (Cohen’s d statistic).

**Region name**	**Ages 0**–**5 years L&R: d**	**Ages 5**–**10 years L&R: d**	**Ages 10**–**15 years L&R: d**	**Ages 15**–**20 years L&R: d**	**MAX ABS(d)**
Inferior lateral ventricle	**L (1.6361) R (1.0511)**	**L (1.9304) R (1.602)**	**L (3.355) R (2.4638)**	**L (2.6248) R (2.0848)**	3.354954938
Lateral Ventricle	**L (1.5806) R (1.4642)**	**L (1.4572) R (1.087)**	**L (3.103) R (1.6137)**	**L (1.9638) R (1.3325)**	3.102984938
Putamen	L (0.42427) R (0.40042)	**L (1.7656) R (1.9786)**	**L (1.3133) R (1.6151)**	**L (2.137) R (2.8152)**	2.815190181
Choroid plexus	**L (1.094) R (1.0546)**	**L (1.9481) R (1.7172)**	**L (1.8626) R (2.0346)**	**L (1.7839) R (2.368)**	2.368035517
Superior part of the precentral sulcus GM	L (0.043307) R (0.0021641)	L (0.23511) R (**−**0.26355)	**L (2.1448)** R (0.46673)	L (0.72085) R (**−**0.18815)	2.144795688
Parahippocampal gyrus GM	L (0.41114) R (0.21504)	L (0.63967) **R (1.2327)**	**L (2.1085)** R (1.134)	**L (1.8185) R (1.5105)**	2.108475198
Amygdala	L (0.19687) R (0.073639)	**L (1.6048) R (1.7743)**	**L (1.6402) R (1.8835)**	**L (1.441) R (1.8243)**	1.883528254
Suborbital sulcus GM	L (0.14062) R (0.29181)	**L (1.2154) R (1.072)**	L (0.45888) R (0.34581)	**L (1.8813)** R (0.70243)	1.881342574
Caudate	**L (0.88451) R (0.91633)**	**L (1.1535) R (1.2114)**	**L (1.5138) R (1.6433)**	**L (1.356) R (1.782)**	1.78203514
Cerebellum WM	L (**−**0.69793) **R (−1.043)**	L (**−**0.84467) R (**−**1.3298)	L (**−**1.1744) **R (−1.5686)**	**L (−1.4275) R (−1.7114)**	1.711363642
Precuneus GM	L (0.16254) R (0.040277)	**L (0.98306)** R (0.84264)	L (0.34212) R (0.72068)	**L (1.6821) R (1.2114)**	1.68214039
Medial occipito**−**temporal sulcus (collateral sulcus) and lingual sulcus GM	L (0.2656) R (**−**0.13837)	L (0.66787) R (0.60091)	**L (1.6152)** R (**−**0.076483)	**L (1.653)** R (0.47595)	1.652991797
Rostral middle frontal GM	L (0.5152) R (0.22144)	L (0.59682) **R (1.0348)**	L (**−**0.24158) R (0.68666)	L (1.2273**) R (1.6494)**	1.649447563
Paracentral WM	L (**−**0.69885) R (**−**0.44313)	L (**−**0.70808) R (**−**0.76241)	L (**−**1.2581) **R (−1.6238)**	L (**−**1.2043) R (**−**1.2937)	1.623784474
Perirhinal GM	L (0.084224) R (0.19062)	L (0.94858**) R (1.4923)**	L (0.95169) **R (1.5377)**	**L (1.371) R (1.5577)**	1.557671423
Anterior corpus callosum	**1.0899**	**1.5023**	**1.3766**	**1.476**	1.502308636
Subcortical GM	0.31442	**1.4633**	0.93052	**1.4945**	1.49452523
Fronto-marginal gyrus (of Wernicke) and sulcus GM	L (0.18326) R (**−**0.10973)	L (0.15527) R (0.73925)	L (1.1226) R (0.69768)	L (1.4575) R (1.4146)	1.457492237
Brainstem	**−1.0097**	−0.72631	−1.084	**−1.4416**	1.441629344
Cortical GM	L (0.024861) R (**−**0.11924)	L (0.71279) R (0.7526)	L (0.17104) R (0.086939)	**L (1.4414)** R (1.3229)	1.441358328
Inferior part of the precentral sulcus GM	L (0.61515) R (0.51061)	L (0.78905) **R (0.99835)**	**L (1.3161) R (1.4209)**	L (1.0301) R (0.86118)	1.420878876
Cerebellar cortex	**L (−1.1979) R (−1.2785)**	**L (−1.3011) R (−1.06)**	**L (−1.3491) R (−1.2879)**	L (**−**1.3086) **R (−1.4136)**	1.41362298
Intraparietal sulcus (interparietal sulcus) and transverse parietal sulci GM	L (0.15995) R (**−**0.086767)	L (0.52677) R (0.12446)	L (**−**0.074757) R (0.94851)	L (1.142) **R (1.4107)**	1.410722414
Brodmann’s area 44 GM	L (0.58412) R (0.38512)	**L (1.0097) R (0.81974)**	L (0.72108) R (**−**0.47666)	**L (1.4021)** R (0.084598)	1.402145386
Superior parietal GM	L (**−**0.19019) R (**−**0.32627)	L (0.14996) R (0.10023)	L (0.33634) R (0.34974)	**L (1.3998)** R (1.1573)	1.399759992
Total cortical GM	−0.048252	0.73952	0.12946	**1.3927**	1.392675827
Entorhinal GM	L (**−**0.068662) R (**−**0.069394)	**L (0.80894) R (1.3459)**	L (0.25628) **R (1.3851)**	L (0.82795) R (0.73824)	1.385095658
Middle temporal GM	L (**−**0.06991) R (**−**0.0886)	L (0.53113) R (0.29518)	L (0.32846) R (**−**0.015145)	**L (1.3846)** R (0.6359)	1.384604341
Precentral GM	L (0.068442) R (**−**0.15049)	L (0.719) R (0.30293)	**L (1.3809)** R (**−**0.11327)	L (0.99009) R (0.39752)	1.380851032
Pars orbitalis GM	L (**−**0.13727) R (0.33074)	L (0.50323) R (0.54924)	L (0.46795) R (0.49176)	L (0.62531) **R (1.3763)**	1.376254549
Temporal pole GM	L (**−**0.0074254) R (**−**0.061858)	L (0.68379) **R (1.3638)**	L (0.58655) R (0.62445)	L (0.42533) R (0.41785)	1.363760336
Insula WM	L (**−**0.10744) R (**−**0.27048)	L (**−**0.40482) R (**−**0.62411)	L (**−**0.70731) R (**−**0.65304)	L (**−**0.55856**) R (−1.3453)**	1.345270221
Lateral occipital GM	L (0.21378) R (**−**0.1679)	L (0.62426) R (0.6965)	L (0.30571) R (0.55768)	L (1.1443) R (1.3232)	1.323232717
Inferior temporal sulcus GM	L (0.32201) R (0.17369)	L (0.31095) R (0.62536)	L (0.88235) R (0.40656)	L (1.3028) R (1.2684)	1.302821804
Brodmann’s area 3a GM	L (**−**0.033975) R (0.24414)	**L (1.0877)** R (0.79785)	L (0.93071) R (0.21018)	L (1.0561) R (1.2988)	1.298763577
Pallidum	L (0.1839) R (0.082274)	**L (1.2791) R (1.1305)**	L (0.69178) R (0.73723)	L (0.63559) R (0.84946)	1.279149736
Superior frontal WM	L (**−**0.32132) R (**−**0.54434)	L (**−**0.4011) R (**−**0.30596)	L (**−**0.65164) R (**−**0.52922)	L (**−**1.265) R (**−**0.79125)	1.264989949
Frontal pole GM	L (**−**0.056314) R (**−**0.18886)	L (0.39103) R (0.68647)	L (**−**0.50156) R (**−**0.50179)	L (0.76575) R (1.2479)	1.247886805
Inferior segment of the circular sulcus of the insula GM	L (**−**0.35994) R (**−**0.46918)	L (**−**0.2484) R (**−**0.63868)	L (**−**1.2454) R (**−**0.89306)	L (**−**0.49113) R (**−**0.80345)	1.245430805
Posterior corpus callosum	**1.2325**	**0.96196**	1.1431	0.82491	1.232473663
Lateral aspect of the superior temporal gyrus GM	L (**−**0.26897) R (**−**0.59274)	L (**−**0.56079) R (**−**0.5578)	L (**−**0.58803) R (**−**1.2311)	L (**−**0.18972) R (**−**0.44347)	1.231065741
Postcentral WM	L (**−**0.55764) R (**−**0.74361)	L (**−**0.50421) R (**−**0.57167)	L (**−**0.80253) R (**−**1.2194)	L (**−**0.64586) R (**−**0.77644)	1.219409271
Transverse frontopolar gyri and sulci GM	L (**−**0.14561) R (**−**0.0939)	L (0.5168) **R (1.1173)**	L (**−**0.23547) R (**−**0.47321)	L (1.1832) R (1.1122)	1.117279157
Middle frontal sulcus GM	L (0.46209) R (0.47663)	**L (1.0595)** R (0.9092)	L (0.048516) R (0.48844)	L (0.42689) R (0.85167)	1.059486009
Brodmann’s area 3b GM	L (**−**0.085189) R (0.13501)	**L (1.0379)** R (0.86403)	L (0.68063) R (0.17709)	L (0.68546) R (0.85308)	1.037881175
Central sulcus (Rolando’s fissure) GM	L (**−**0.1311) R (0.059043)	**L (1.0145) R (1.0258)**	L (0.55) R (**−**0.054695)	L (0.83131) R (0.73035)	1.025831833
Third ventricle	**1.0037**	0.48892	0.91313	1.1851	1.003728642
Occipital pole GM	L (0.56066) R (0.06348)	L (0.91735) **R (0.99864)**	L (0.56553) R (0.43693)	L (0.65984) R (1.1901)	0.998637388
Inferior temporal gyrus GM	L (0.13636) R (0.26431)	L (0.35303) **R (0.9807)**	L (**−**0.51814) R (**−**0.053426)	L (0.94713) R (0.88104)	0.980702759
Optic chiasm	0.48811	**0.9659**	0.56354	1.0442	0.965901131
Subparietal sulcus GM	L (0.14009) R (0.13493)	**L (0.96306)** R (0.85858)	L (0.71761) R (**−**0.0089489)	L (0.22741) R (0.63616)	0.963060254
Rostral anterior cingulate GM	L (**−**0.55121) R (**−**0.75501)	L (**−**0.95204) R (**−**0.77911)	L (**−**0.87413) R (**−**0.61322)	L (**−**0.8092) R (**−**0.29236)	0.952036615
Ventral diencephalon	L (0.18328) R (0.2278)	L (0.88711) R (0.92695)	L (0.32581) R (0.71464)	L (**−**0.20781) R (0.15866)	0.926951994
Paracentral lobule and sulcus GM	L (0.022597) R (0.1339)	L (0.90257) R (0.61371)	L (0.024697) R (0.059733)	L (0.26181) R (0.75372)	0.902566782
Inferior frontal sulcus GM	L (0.30856) R (0.37135)	L (0.4197) R (0.90008)	L (**−**0.51999) R (0.85469)	L (1.0593) R (0.61716)	0.900077596
Fourth ventricle	0.79775	0.42461	0.36745	0.6556	0.797752661
Cerebral spinal fluid	0.79405	−0.099041	−0.39824	0.092083	0.794049677

Abbreviations/Symbols: GM = gray matter; WM = white matter; R = right; L = Left; d = Cohen’s d statistic. Bold entries indicate a statistically significant finding after multiple comparisons correction on at least one available FreeSurfer atlas.

**Fig. 1:**
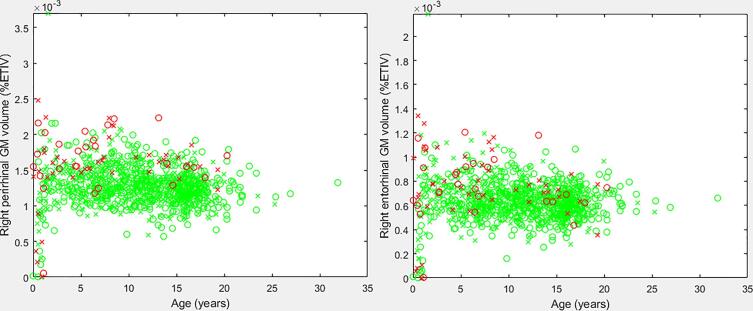
Scatter plots of the right perirhinal (left pane) and right entorhinal (right pane) volumes (%ETIV). Green samples represent neurotypical participants, red samples represent DS participants. X represents a male, O a female. (For interpretation of the references to colour in this figure legend, the reader is referred to the web version of this article.)

The age-dependent d statistic analysis yielded a variety of measurements that may aid in understanding the anatomical presentation of the DS brain. Table 2, Table 3 present the leading measurements organized by Cohen’s d statistic, with the highest d values being found at the top of the table. Thus, the inferior lateral ventricle exhibits the largest group wise difference between DS and neurotypical participants (ages 10–15), followed by the lateral ventricle (ages 10–15), and so on. We presented the raw Cohen’s d statistic as opposed to the adjusted statistic for ease of comparison with future studies. Fig. 1 provides a scatter plot of our findings pertaining to the perirhinal and entorhinal regions (%ETIV). Supplementary Figs. S1 through S6 provide scatter plots of our leading findings from Table 2, Table 3.

## 4 Discussion

We performed a large-scale volumetric analysis of structural MRI examinations in DS and neurotypical individuals and demonstrated group-wise differences in various brain regions such as greater volumes in the perirhinal cortex, entorhinal cortex, choroid plexus (CP), and Brodmann’s areas (BA) 3a, 3b, and 44, as a percentage of estimated total intracranial volume (%ETIV), in the context of smaller absolute brain volumes in the DS cohort. Our analysis also demonstrated reduced volumes (%ETIV) in the WM of the cuneus, the paracentral lobule, the postcentral gyrus, and the supramarginal gyrus. To the best of our knowledge, this is the first time that volumetric measurements for these brain regions have been reported in the literature for individuals with DS. Our analysis also included four age cohorts (0–5, 5–10, 10–15, and 15–20 year olds), and a general trend was observed whereby the youngest age cohort (0–5 year olds) tended to exhibit the smallest effect sizes (d statistics closest to zero). This could imply that the structural abnormalities identified in this study progress through childhood/adolescence, thus there may be value in conducting future studies tracking patients in a longitudinal study design, rather than the cross sectional design of this research.

The perirhinal and entorhinal cortices are located in the medial temporal lobe and form important connections between the neocortex and the hippocampus suggesting that these brain regions play an important role in memory (Van Hoesen et al., 1991). Specifically, the perirhinal and parahippocampal cortices provide sensory input to the hippocampus through entorhinal connections and receive output from the hippocampus through the entorhinal pathway (Burwell, 2000, Insausti et al., 1997, Lavenex and Amaral, 2000). We observed increased volumes (%ETIV) in the perirhinal cortex, consisting of BA 35 and 36 (Insausti et al., 1998), and the entorhinal cortex, consisting of BA 28 (Juottonen et al., 1998), in DS. Numerous animal model studies have demonstrated that lesions in the perirhinal cortex lead to significant impairments in recognition memory (Meunier et al., 1993, Wan et al., 1999, Zola-Morgan et al., 1989) and lesions in the entorhinal cortex have been associated with memory deficits (Buckmaster et al., 2004, Staubli et al., 1986). Volumetric reductions in the perirhinal and entorhinal cortices have also been reported in individuals with AD, including a 27% reduction in the perirhinal cortex (Juottonen et al., 1998) and a 40% reduction in the entorhinal cortex in individuals with AD (Gómez-Isla et al., 1996, Juottonen et al., 1998). Adults with DS are at increased risk for AD (Torr et al., 2010). There is significant overlap between the neuropathology of AD and adults with DS over the age of 40 years. These features include the development of senile plaques and neurofibrillary tangles (Folin et al., 2003, Head et al., 2012, Wisniewski et al., 1985). It is hypothesized that Alzheimer’s neuropathology is largely caused by the overexpression of the amyloid precursor protein (APP) located on chromosome 21 because APP produces amyloid ß protein, the main component of senile plaques (Hardy and Higgins, 1992). Observed abnormalities in DS from our study include the perirhinal and entorhinal cortices, which may be associated with increased prevalence of AD in DS as these brain regions play an important role in memory and with this study’s findings, abnormalities of these regions have now been reported in both conditions. Although the cohort we have investigated is younger than the age ranges in which AD typically develops, we have observed early changes in the perirhinal and entorhinal cortices, regional abnormalities of which have been associated with AD. It should also be noted that many of our findings indicate increased brain growth deviations with age in the DS cohort relative to our neurotypical participants. Recent findings from fetal brain MRI of patients with DS indicate that neurodevelopmental abnormalities can be detected in vivo (Tarui et al., 2020, Patkee et al., 2020). These fetal based findings combined with the data in this study are potentially supportive of the theory that developmental brain abnormalities in DS increase with patient age. Thus, it is possible that, for instance, perirhinal developmental abnormalities may increase with age, and potentially contribute to memory problems and the common development of AD in DS, a subject for future research.

Our results include increased volumes (%ETIV) in BA 44 in DS, part of Broca’s area in the posterior portion of the left inferior frontal gyrus (Hagoort, 2005, Schnur et al., 2009). Broca’s area is well known as an important brain region for speech production. Individuals with DS experience difficulties in speech and language with expressive language appearing to be weaker than receptive language (Caselli et al., 1998, Chapman et al., 2002, Laws and Bishop, 2003, Martin et al., 2009). Abnormalities in BA 44 (i.e. Broca’s area) may be associated with known speech production deficits in DS.

Our data showed absolute and relative (%ETIV) volume increases of the CP in individuals with DS. The CP is a highly vascularized tissue that acts like a “kidney” for the brain as it maintains the chemical stability of the CSF through several functions including the secretion, production, and cleansing of the CSF, the protection of the brain through increased buoyancy, and the secretion of certain proteins (Spector and Johanson, 1989). The epithelial layer of the CP also forms the blood-CSF barrier (Kunis et al., 2013). The CPs are located within brain ventricles (Strazielle and Ghersi-Egea, 2000). Our data and previous studies have reported enlarged ventricles in DS (Beacher et al., 2010, Frangou et al., 1997, Pearlson et al., 1998, Raz et al., 1995, White et al., 2003). Because the CP is involved in CSF production within the ventricles, it is plausible that increases in CP volume is directly related to known increases in ventricular volumes.

BA 1, 2, and 3 compose the primary somatosensory cortex in the postcentral gyrus, a brain region responsible for processing somatic sensations such as touch, proprioception, nociception, and temperature (Webb, 2017). We observed increased volumes (%ETIV) in BA 3a and 3b. These areas are of particular importance as BA 3b receives most of its input directly from the thalamus and performs initial processing, and BA 3a plays an important role in motor activity (Borich et al., 2015). Sensory information is essential for basic motor control/functionality as it is used to initiate movement by selecting and activating appropriate motor control structures, it is used during movement for triggering successive motion patterns, and it is continuously used as feedback or as a time base for a motor program (Cruse et al., 1990). There has been evidence that abnormal processing by the primary somatosensory cortex contributes to deficits seen in disorders characterized by motor dysfunction, such as Parkinson’s disease, dystonia, and ataxia (Borich et al., 2015). Although DS is not typically classified as a motor control disorder, it is characterized by specific motor deficits such as increased motion and reaction times, balance and posture deficits, and simultaneous contractions of agonist and antagonist muscle pairs (Galli et al., 2008), with motor coordination and hypotonia representing the main motor issues faced by DS patients (Capio, et al., 2018). The numerous motor deficits may, at least in part, be related to the observed abnormalities in BA 3a and 3b. Numerous studies have also reported that the primary somatosensory cortex plays an important role in pain perception (Backonja, 1996, Bushnell et al., 1999, Vierck et al., 2013). Research suggests that pain perception is abnormal in individuals with DS who experience an increased sensitivity to pain (McGuire and Defrin, 2015, Valkenburg et al., 2015). Thus, abnormalities in the primary somatosensory cortex may be associated with increased pain sensitivity in individuals with DS.

Previous studies have reported gray matter volume reductions in the left cuneus (White et al., 2003), the paracentral lobule (Carducci et al., 2013, Matthews et al., 2016), and the postcentral gyrus (Carducci et al., 2013, Teipel et al., 2004). To the best of our knowledge, this is the first time WM volume (%ETIV) reductions have been reported for the cuneus, the paracentral lobule, the postcentral gyrus, and the supramarginal gyrus. Abnormal WM volumes in these regions may implicate structural connectivity abnormalities between those same region’s GM and other areas of the brain. In effect, the anteromedial cuneus plays an important role in visual perception as it interacts with the primary visual cortex (V1) and modifies information transferred via V1 to extrastriate cortices (Vanni et al., 2001). The paracentral lobule has been linked to motor control of the contralateral lower limb (Laplane et al., 1977) and so reductions in the WM of the paracentral lobule may be linked to motor deficits in DS, including balance and postural issues (Galli et al., 2008). WM reductions in the postcentral gyrus may be related to sensory deficits in infants and children with DS (Chen and Fang, 2005, Hayes and Batshaw, 1993) as lesions in this area have been associated with sensory loss (Corkin et al., 1970). Finally, the supramarginal gyrus is thought to be involved in phonological aspects of word processing as it forms connections to auditory association regions in the posterior supratemporal plane and the posterior inferior frontal gyrus (Stoeckel et al., 2009). Abnormalities in the WM of this region may contribute to language difficulties in individuals with DS (Roberts et al., 2007).

Although the majority of our study’s primary findings are based on volumetric (%ETIV) abnormalities, our analysis also included absolute volumetric regional assessments. Our data displayed volumetric increases not only in the relative (%ETIV) volumes, but also in absolute volumes of several regions including the CP. Furthermore, our data also demonstrated absolute volumetric decreases in the WM of the cuneus, paracentral lobule, postcentral gyrus, and supramarginal gyrus. Our data confirmed findings from previous studies such as decreased absolute volumes of the whole brain (Aylward et al., 1999, Aylward et al., 1997, Beacher et al., 2010, Frangou et al., 1997, Kates et al., 2002, Menghini et al., 2011, Pearlson et al., 1998, Pinter et al., 2001a, Pinter et al., 2001b, Śmigielska-Kuzia et al., 2011, Weis et al., 1991), the planum temporale (Frangou et al., 1997), the cerebrum (Jernigan et al., 1993, Jernigan and Bellugi, 1990, Kates et al., 2002, Raz et al., 1995), the cerebral cortex (Weis et al., 1991), the brainstem (Carducci et al., 2013, Fujii et al., 2017, Jernigan and Bellugi, 1990), decreased absolute and (%ETIV) volumes of the hippocampus (Aylward et al., 1999, Carducci et al., 2013, Pearlson et al., 1998, Pinter et al., 2001a, Raz et al., 1995, Śmigielska-Kuzia et al., 2011, Teipel et al., 2003), the cerebellum (Beacher et al., 2010, Carducci et al., 2013, Jernigan et al., 1993, Jernigan and Bellugi, 1990, Pinter et al., 2001b, Raz et al., 1995, Wang et al., 1992, Weis et al., 1991, White et al., 2003), the insula (Carducci et al., 2013, White et al., 2003), and the cingulate gyri (Carducci et al., 2013, Raz et al., 1995, White et al., 2003). Our data also confirmed increased absolute and (%ETIV) volumes in the lateral ventricles (Beacher et al., 2010, Frangou et al., 1997, Pearlson et al., 1998, White et al., 2003), the third ventricle (Raz et al., 1995), the putamen (Aylward et al., 1997, Beacher et al., 2010, Jernigan et al., 1993), and the parahippocampal gyrus (Carducci et al., 2013, Raz et al., 1995, White et al., 2003).

In order to assess the potential effects of rapid early growth in the 0–2 year age range, we repeated the %ETIV analysis on only the 0–5 year old cohort, divided into two sub cohorts: 0–2 years old (rapid growth period), and 2–5 years old. Our findings indicate that the leading biomarkers exhibiting differences between the 0–2 and the 2–5 year olds are predominantly found in the 2–5 year old cohort as compared with the 0–2 year olds. The leading biomarkers for the 2–5 year olds were the left lateral ventricle volume, right lateral ventricle volume, posterior corpus callosum volume, left choroid plexus volume, anterior corpus callosum volume, right inferior lateral ventricle volume, right choroid plexus volume, right cerebellar cortex volume, and the right parahippocampal volume. These are very consistent with the findings in Table 3 (0–5 year old cohort), with the exception of the right parahippocampal gyrus volume which exhibits a much larger cohen’s d statistic (d = 1.3) in the 2–5 year old age group than was reported in the 0–5 year old group (d = 0.2), potentially implying that parahippocampal gyral abnormalities in Down Syndrome are detectable during early childhood. The leading biomarkers for the 0–2 year olds were the left inferior lateral ventricle volume, right cerebellar cortex volume, left lateral ventricle volume, left cerebellar cortex volume, right lateral ventricle volume, posterior corpus callosum volume, anterior corpus callosum volume, and right caudate volume. These leading findings from the 0–2 year old age group are consistent with those reported in the 0–5 year old age group in Table 3.

The strengths and limitations of our study design has been previously discussed in detail (Levman et al., 2019, Levman et al., 2018, Levman et al., 2017). The main strength of our study is the large cohort of neurotypical participants with which the DS participants could be compared. This provides a statistically reliable baseline from which to assess DS related differences. Another strength is that our dataset includes many examinations from the 0–5 year age range, a cohort that is minimally studied in the scientific literature. Limitations include the variability in imaging parameters due to variations in the pulse sequences employed and the small sample size for the DS group, especially in the 10–15 age group (15 participants) and the 15–20 age group (9 participants). We had insufficient samples in the DS group to create reliable multivariable regression models that control for the effects of pulse sequence variability. Additional limitations include the retrospective nature of the analysis (patients were referred to MRI for a clinical reason and therefore may display more extreme characteristics of DS), the lack of neurocognitive function assessment of individuals with DS (e.g. intelligence quotient (IQ) information), lack of detailed patient interviews providing complete assessments of comorbidities, and an imbalanced pool of participants. The Bonferroni correction was selected because it is the strictest accepted correction for the multiple comparisons problem. As such, using it in this situation helps to limit the reporting of spurious findings in this analysis. However, it should also be noted that this can potentially result in us reporting fewer statistically significant findings in our main reported tables (see bold entries in Table 2, Table 3). In short, while the Bonferroni correction results in a shorter list of statistically significant findings, it also helps limit the amount of spurious findings that we report as statistiscally significant, which in the context of real-world clinical data and natural variability found in both datasets, was deemed a desirable analytic option to help prevent the reporting of findings that will not hold in future studies. Finally, FreeSurfer is not optimized for our youngest participants in the 0–8 months-old age range leading to uncertainty in the reliability of these results. Research aimed at overcoming FreeSurfer’s reliability and applicability in younger populations is ongoing (de Macedo Rodrigues et al., 2015, Zollei et al., 2017), and will be incorporated into future work.

Our results indicate group-wise differences in the volumes of various brain regions. Our main findings include volume increases (%ETIV) in the perirhinal cortex, entorhinal cortex, CP, and BA 3a, 3b, and 44, and volume decreases (%ETIV) in the WM of the cuneus, the paracentral lobule, the postcentral gyrus, and the supramarginal gyrus. These volumetric brain abnormalities may contribute to some of the phenotypic symptoms exhibited by individuals with DS. We also clinically validated findings previously discussed in the literature such as volumetric decreases in the whole brain, the planum temporale, the cerebrum, the cerebral cortex, the brainstem, the hippocampus, the cerebellum, the insula, and the cingulate gyri and volumetric increases in the ventricles, the putamen, and the parahippocampal gyrus. Future work might benefit from investigating whether the regional structural abnormalities identified in this study may be related to functional symptoms in DS, by prospectively analyzing a DS population with detailed clinical data available for each patient. Future work will incorporate a variety of additional neuroimaging and analytical techniques such as diffusion tensor imaging, functional MRI, and multivariate machine learning, to help better elucidate our understanding of the abnormal neurodevelopment associated with DS.

## Funding

The authors would like to thank Dr. Henry Feldman, Principal Biostatistician at Boston Children’s Hospital for advice on conducting statistical analyses. This work was supported by the National Institutes of Health (grant numbers R01HD078561, R21MH118739, R03NS091587, R21HD098606) to ET; Natural Science and Engineering Research Council of Canada's Canada Research Chair grant (grant number 231266) to JL, Natural Science and Engineering Research Council of Canada Discovery Grant to JL, a Canada Foundation for Innovation and Nova Scotia Research and Innovation Trust infrastructure grant (R0176004) to JL, a St. Francis Xavier University research startup grant to JL (grant number R0168020), a St. Francis Xavier University UCR grant to JL, and a Nova Scotia Health Research Foundation Scotia Scholars Award to BM. JL is founder of Time Will Tell Technologies, Inc., the rest of the coauthors have no conflicts of interest to report.

## CRediT authorship contribution statement

**Bernadette McCann:** Formal analysis, Investigation, Methodology, Validation, Writing – original draft, Writing – review & editing. **Jacob Levman:** Conceptualization, Data curation, Formal analysis, Funding acquisition, Investigation, Methodology, Project administration, Supervision, Validation, Writing – original draft, Writing – review & editing. **Nicole Baumer:** Formal analysis, Writing – review & editing. **Melanie Y. Lam:** Supervision, Writing – review & editing. **Tadashi Shiohama:** Conceptualization, Writing – review & editing. **Liam Cogger:** Methodology, Validation. **Allissa MacDonald:** Writing – original draft. **Prahar Ijner:** Software. **Emi Takahashi:** Conceptualization, Funding acquisition, Project administration, Supervision, Writing – review & editing.
